# Multiparametric MRI characterization of level dependent differences in lumbar muscle size, quality, and microstructure

**DOI:** 10.1002/jsp2.1079

**Published:** 2020-02-03

**Authors:** David B. Berry, Ana E. Rodriguez‐Soto, Erin K. Englund, Bahar Shahidi, Callan Parra, Lawrence R. Frank, Karen R. Kelly, Samuel R. Ward

**Affiliations:** ^1^ Department of Bioengineering University of California San Diego California; ^2^ Department of Nanoengineering University of California San Diego California; ^3^ Department of Radiology University of California San Diego California; ^4^ Department of Orthopaedic Surgery University of California San Diego California; ^5^ Department of Exercise and Nutritional Sciences San Diego State University San Diego California; ^6^ Warfighter Performance Department Naval Health Research Center San Diego California

**Keywords:** diffusion tensor imaging, fatty infiltration, lumbar spine, MRI, skeletal muscle

## Abstract

Magnetic resonance imaging (MRI) is a diagnostic tool that can be used to noninvasively assess lumbar muscle size and fatty infiltration, important biomarkers of muscle health. Diffusion tensor imaging (DTI) is an MRI technique that is sensitive to muscle microstructural features such as fiber size (an important biomarker of muscle health), which is typically only assessed using invasive biopsy techniques. The goal of this study was to establish normative values of level‐dependent lumbar muscle size, fat signal fraction, and restricted diffusion assessed by MRI in a highly active population. Forty‐two active‐duty Marines were imaged using a (a) high‐resolution anatomical, (b) fat‐water separation, and (c) DT‐MRI scan. The multifidus and erector spinae muscles were compared at each level using two‐way repeated measures ANOVA. Secondary analysis included Three dimensional (3D) reconstructions to qualitatively assess lumbar muscle size, fatty infiltration, and fiber orientation via tractography. The erector spinae was found to be larger than the multifidus above L5, with lower fat signal fraction above L3, and a less restricted diffusion profile than the multifidus above L4, with this pattern reversed in the lower lumbar spine. 3D reconstructions demonstrated accumulations of epimuscular fat in the anterior and posterior regions of the lumbar musculature, with minimal intramuscular fatty infiltration. Tractography images demonstrated different orientations of adjacent lumbar musculature, which cannot be visualized with standard MRI pulse sequences. The level dependent differences found in this study provide a normative baseline, for which to better understand whole muscle and microstructural changes associated with aging, low back pain, and pathology.

## Graphical abstract

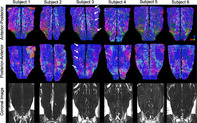

## 1 INTRODUCTION

The posterior muscles of the lumbar spine (erector spinae and multifidus) provide mechanical stability to the lumbar vertebral segments, as well as support the upper trunk.^1^, ^2^, ^3^ Several studies have investigated the structural and compositional properties of the lumbar musculature, in order to better understand its function in healthy and injured populations.^1^, ^4^, ^5^, ^6^, ^7^ Most studies of these muscles are histology based, which is highly invasive, destructive to the tissue, and only provides information about a small fraction of the whole muscle. Additionally, most tissue obtained for histological analysis is typically acquired via biopsy in patients undergoing surgery for lumbar pathology or from cadavers, making it difficult to evaluate control tissue to quantify healthy muscle. These factors have driven interest in developing noninvasive imaging techniques to study and monitor whole lumbar muscle health.

Magnetic resonance imaging (MRI) is a diagnostic tool often used to evaluate anatomic pathology, as well as to obtain quantitative measures of lumbar muscle size, quality (fatty infiltration), and intervertebral disc (IVD) health. Decreased muscle size and increased fatty infiltration—important biomarkers of lumbar muscle health—associated with age, low back pain, and lumbar spinal pathology (ie, IVD degeneration), are often observed with MRI.^4^, ^5^, ^8^, ^9^, ^10^ As the amount of functional contractile tissue decreases due to atrophy or fatty infiltration, the force generating capacity of muscle decreases, which can in turn affect the ability of the posterior lumbar muscles to stabilize the spine. Reductions in these muscle health parameters have been associated with poor prognosis for recovery from pain or pathology.^11^

While whole lumbar muscle changes associated with pathology or low back pain are often observed, there is some evidence that changes in muscle fibers themselves are more discriminative of lumbar spine pathology.^12^ Diffusion tensor imaging (DTI) is an MRI technique which has been shown to be sensitive to features of muscle microstructure.^13^, ^14^, ^15^ DTI measures the restricted diffusion profile of water within a tissue, and as the sarcolemma is believed to be the primary barrier to diffusion, restricted diffusion is thought to be most sensitive to fiber size.^16^, ^17^, ^18^, ^19^ In normal nonedematous muscle, less restricted diffusion is thought to be indicative of increased muscle fiber size.^16^ As fiber size is a crucial biomarker for whole muscle health, there is growing interest in using DTI to monitor changes in muscle microstructural properties associated with injury and disease.^20^

In addition to quantitative measures of muscle microstructure, DTI also allows for the visualization of some macroscopic features of muscle architecture through the use of fiber tractography algorithms. Tractography is a postprocessing technique, where the principle diffusion directions of neighboring voxels are used to create a visual representation of muscle architecture.^21^, ^22^, ^23^ This allows for the approximation of muscle pennation angle and raw (non‐normalized) fiber length,^24^, ^25^ which is difficult, if not impossible, to appreciate with routine MRI.

A few studies have used DTI to assess lumbar microstructure and architecture in healthy patients. However, the majority of these studies suffer from multiple methodologic problems related to data acquisition and processing, which affects overall data quality and reproducibility. Some of these issues include: low spatial resolution,^26^, ^27^ long echo times (reducing overall muscle signal),^26^, ^27^, ^28^ not scanning the entire lumbar musculature,^26^, ^27^, ^28^ arbitrary region of interest (ROI) definitions not based on muscle anatomy,^26^, ^27^ and not accounting for susceptibility‐induced geometric distortions.^27^, ^28^ One DTI based study has used high quality acquisition and postprocessing techniques to study lumbar muscle architecture using tractography.^29^ However, only one subject was evaluated, so normal variations in DTI‐measured lumbar microstructure or architecture were not addressed. Therefore, there is a need for high‐quality, reproducible DTI data in the muscles of the lumbar spine and an understanding of variability in DTI‐based measurements of muscle microstructure and architecture in healthy subjects. The goal of this study was to perform a systematic, level by level evaluation of the size, fat signal fraction, and the restricted diffusion profile of the lumbar muscles in a highly‐active, healthy population (active duty US Marines). The erector spinae and multifidus muscles were directly compared at each level in order to better understand how the physiology and microstructure of these muscles change throughout the entire volume of the lumbar spine. Additionally, Three dimensional (3D) volumetric reconstructions of the lumbar muscles were made in order to qualitatively illustrate how muscle size, fatty infiltration, and fiber orientation change over the entire volume of the lumbar spine in different subjects.

## 2 METHODS

### 2.1 Study design

This was a secondary analysis of existing data that was originally collected to investigate the predictive ability of MRI‐based biomarkers of muscle health on lumbar posture in simulated operational positions in active‐duty Marines.^30^, ^31^

### 2.2 Participants

The University of California, San Diego and Naval Health Research Center Institutional Review Boards approved this study and all Marines gave oral and written consent to participate. Marines were included in this study if they were male, over 18 years of age, and fit to perform their assigned duty. Marines were excluded from this study if they had undergone lumbar spine surgery or had the possibility of shrapnel and/or other metal combat fragments in their bodies. All Marines underwent standard MRI safety screening prior to scanning. All scans were performed between 0400 and 0900.

### 2.3 Magnetic resonance imaging

Marines were scanned supine using a 3T MRI system (GE MR350 Discovery, GE Healthcare, Waukesha, Wisconsin) and a spine array coil. The imaging protocol consisted of (a) a high resolution anatomical scan (fast spoiled‐gradient echo; FSPGR), (b) a fat‐water separation scan (iterative decomposition of water and fat with echo asymmetry and least‐squares estimation; IDEAL),^32^ and (c) a spin‐echo DTI scan using an echo planar acquisition. The DTI sequence was acquired twice, once in the positive and once in the negative phase encoding direction, in order to correct for susceptibility‐induced geometric distortions associated with echo planar acquisition. The scanning parameters are listed in Table 1. Anatomical and DTI scans were acquired in the axial plane. The water‐fat separation scan was acquired in the sagittal plane. All scans were acquired from the superior endplate of L1 to the inferior endplate of S1.

**Table 1:** Scanning parameters for each pulse sequence used in this study

Pulse sequence	TR (ms)	TE (ms)	FoV (cm)	Acquisition and reconstruction matrix	Voxel size (mm)	Slices	NEX	# dir.	*b*‐value (s/mm^2^)	Scan time
FSPGR	5	2.3	32	512 × 512	0.625 × 0.625 × 1	206	3	‐	‐	10:34
IDEAL	1974	160	25.6	256 × 256	1 × 1 × 1	186	1	‐	‐	14:02
DTI	10 000	46	19.2	128 × 128	1.5 × 1.5 × 3	82	1	45	400	7:50 × 2

Abbreviations: avg., averages; dir., directions; DTI, diffusion tensor imaging; FoV, field of view; FSPGR, fast spoiled‐gradient echo; IDEAL, iterative decomposition of water and fat with echo asymmetry and least‐squares estimation; TE, excitation time; TR, repetition time.

### 2.4 Data processing

**Figure 1:**
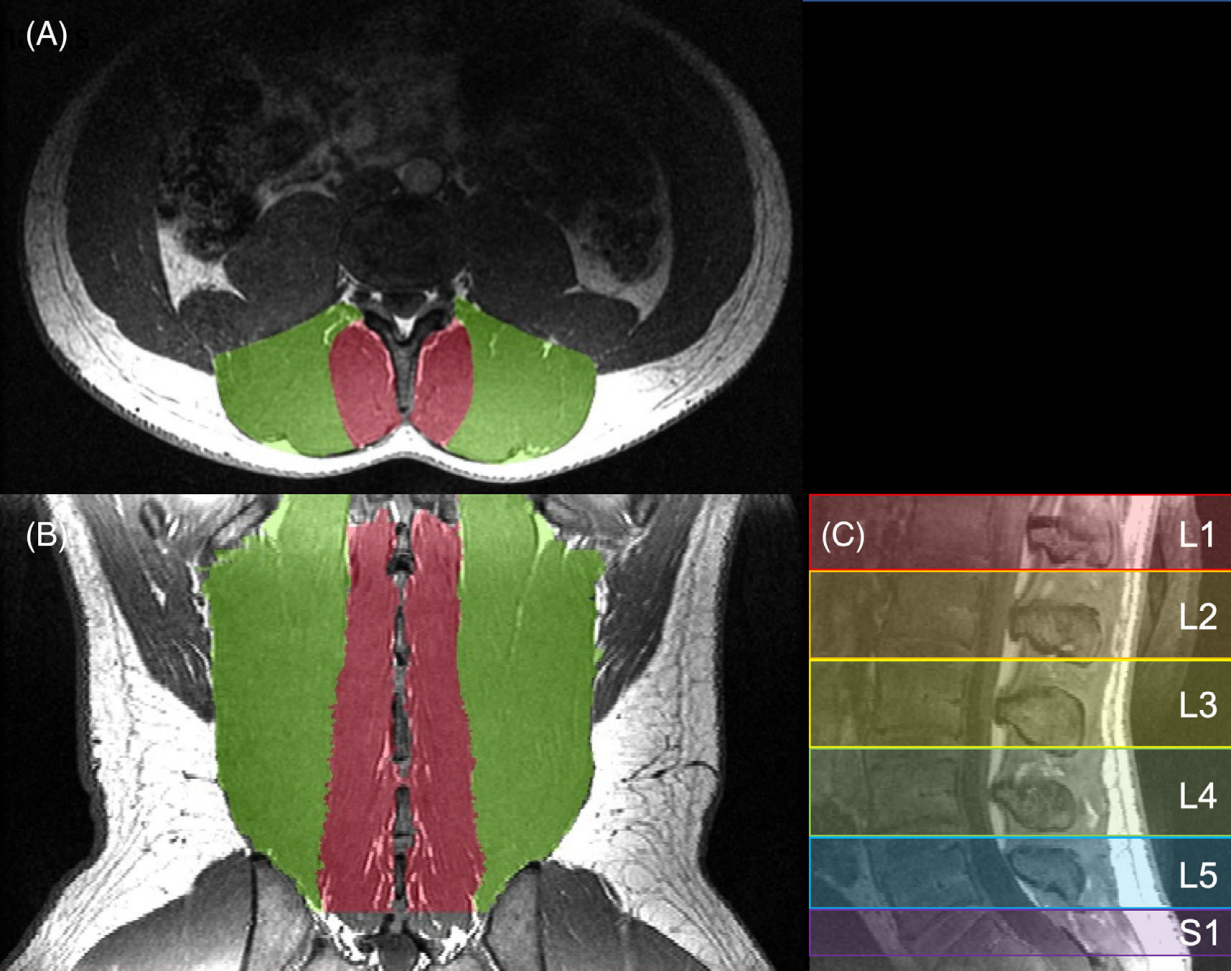
Sample anatomical MRIs used for segmentation of the multifidus (red) and erector spinae (green). A, Segmentations were manually drawn in OsiriX in the axial plane. B, Coronal reformatted MRI of the multifidus and erector spinae muscle masks. C, Mid‐sagittal reformatted MRI demonstrating how level dependent measures were segmented from the midpoint between adjacent vertebrae

**Figure 2:**
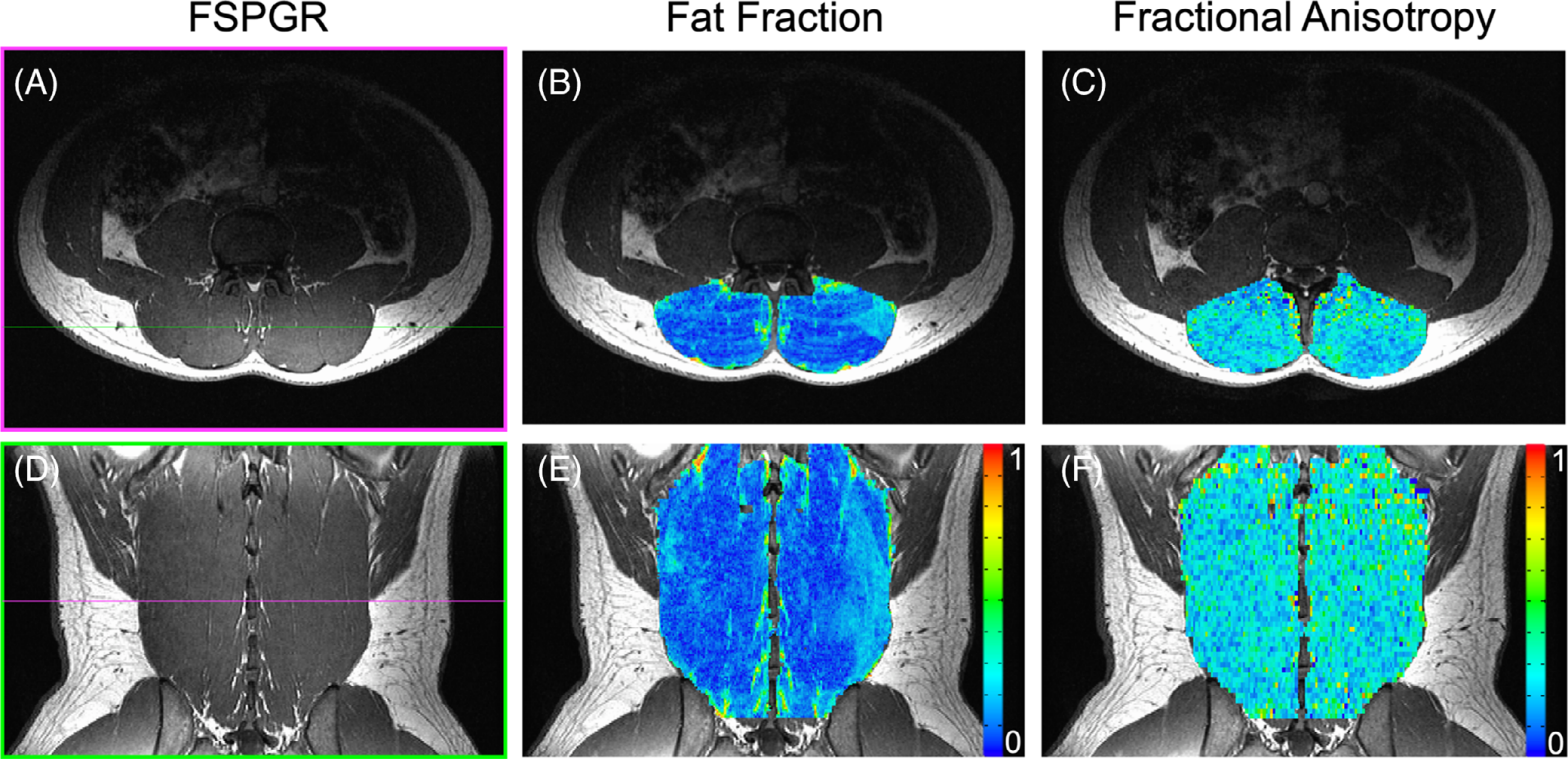
Representative axial (top) and coronal reformatted (bottom) MRIs of the high resolution FSPGR scan (A,D), fat fraction map (B,E), and fractional anisotropy map (C,F) at the L3. The bands of signal in the axial fat fraction map (B) are due to a breathing artifact

ROIs were made using a previously published approach, which has been shown to have high inter‐rater agreement (ICC > 0.928).^33^ High resolution anatomical images were imported into the OsiriX imaging software for segmentation.^34^ Contours of the multifidus and erector spinae group were manually traced from the L1 to S1 lumbar levels (Figure 1A,B). In order to standardize vertebral level‐based comparisons between subjects, ROIs for each level were generated halfway between the endplates of adjacent vertebrae, through the center of each adjacent IVD, over which volume, fat fraction, and diffusion measurements were calculated (Figure 1C). The resulting segmentations were used to generate masks to quantify muscle volume, fat fraction, and diffusion properties of the muscles from each subject (Figure 2). Masks from the high resolution anatomic scan were resampled in order to match the resolution of the fat‐water separation and DTI scans using the Analysis of Functional NeuroImages (AFNI; National Institutes of Health, Bethesda, Maryland) command 3dresample.^35^

Images acquired using the fat‐water separation sequence yielded two sets of images: one where both fat and water MRI signals are in phase, and one where they are out of phase. A water only signal image (S_w_) can be calculated by adding the in phase and out of phase images and dividing by two. A fat only signal (S_f_) image can then be derived by subtracting the water only signal image from the in phase image. Therefore the independent signal contributions of both the fat and water signals can be isolated within each voxel. This can then be used to calculate the fat signal fraction within a voxel:

$$\mathrm{fat}\;\text{signal fraction}=\frac{S_{f}}{S_{f}+S_{w}}$$

Phase and distortion correction from DTI images were performed using TOPUP.^36^, ^37^ The diffusion tensor was calculated using the AFNI command 3dDWItoDT. The eigenvalues (*λ*_1,2,3_), mean diffusivity (MD), radial diffusivity (RD), and fractional anisotropy (FA) were calculated. The eigenvalues of the diffusion tensor describe the magnitude of diffusion along their respective eigenvectors. The primary eigenvalue (*λ*_1_) describes diffusion along the primary axis of muscle fibers, and the second and third eigenvalues (*λ*_2_, *λ*_3_) describe diffusion orthogonal to *λ*_1_. MD is the average of all the eigenvalues (*λ*_1,2,3_) and describes overall mean squared displacement, in units of mm^2^/s. RD is the average of *λ*_2_ and *λ*_3_ and is thought to be sensitive to diffusion orthogonal to the primary axis of a muscle fiber. FA is a measure of the variance of the eigenvalues and describes diffusion anisotropy from 0 (perfectly isotropic) to 1 (perfectly anisotropic). Since the sarcolemma is thought to be the main barrier to diffusion, diffusion is greater longitudinally (*λ*_1_) than radially (*λ*_2_, *λ*_3_) across muscle fibers.^13^, ^14^, ^16^

Muscle size, and the distribution of fat within a muscle varies throughout the volume of a muscle.^4^ In order to visualize the distribution of muscle size and fatty infiltration in the lumbar musculature, 3D reconstructions were made using the volume rendering module in 3D Slicer.^38^ Tractography models were generated in Diffusion Toolkit^39^ and visualized in TrackVis. An angular threshold of 15° was used as termination criteria and an interpolated streamline propagation algorithm was used.

### 2.5 Statistical analysis

To identify level dependent differences between the multifidus and erector spinae muscles, all muscle structure measurements were compared using a two‐way repeated‐measures analysis of variance (ANOVA) with post hoc Sidak tests (factors: lumbar level, erector spinae/multifidus). Statistical analysis was performed using the SPSS Statistics software (version 25.0, IBM, Armonk, New York). The threshold for significance (*α*) was set to .05 for all analyses. Data are reported as mean ± SD.

## 3 RESULTS

### 3.1 Volunteer demographics

Forty‐three Marines volunteered for this study. One subject withdrew due to claustrophobia. Due to incomplete data acquisitions (7) or breathing/motion artifacts (1), the DTI data sets of eight subjects were not included in the DTI analysis. Therefore, volume and fat signal fraction data was collected in 42 Marines (age = 26.9 ± 6.4 years, height = 178.0 ± 7.1 cm, weight = 81.8 ± 9.9 kg, BMI = 25.8 ± 2.9 kg/m^2^), and DTI data was collected in 34 Marines (age = 26.8 ± 6.7 years, height = 178.1 ± 7.6 cm, weight = 81.1 ± 9.6 kg, BMI = 25.6 ± 2.7 kg/m^2^).

### 3.2 Level and muscle dependent differences in lumbar muscle size and quality

**Figure 3:**
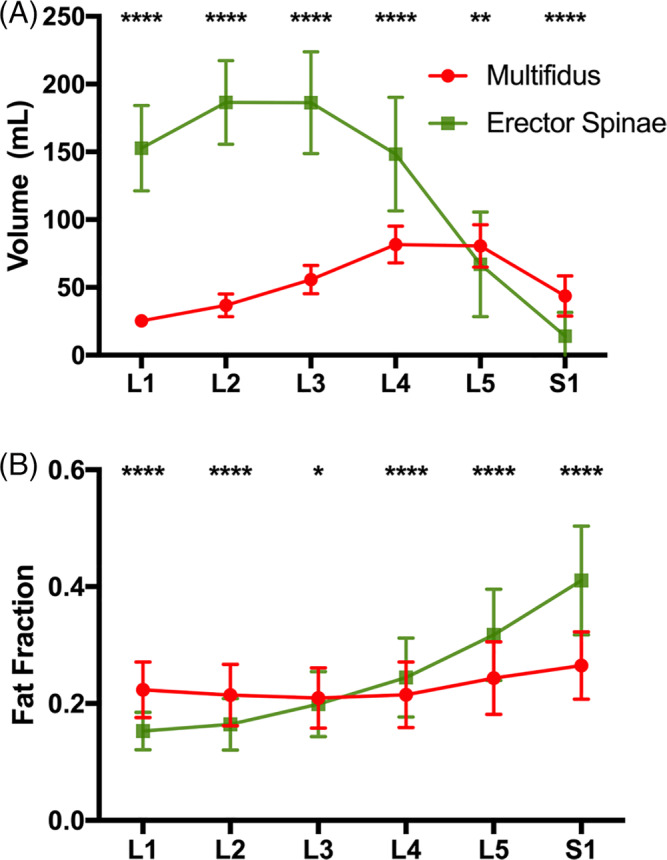
A, Muscle volume and B, fat signal fraction at each lumbar spine level (L1‐S1) for the multifidus (red) and erector spinae (green) muscles. Asterisks indicate significant differences between muscles at each level (*P < .05; **P < .01; ***P < .001; ****P < .0001)

A main effect of muscle (*P* < .0007), vertebral level (*P* < .0001), and an interaction between muscle and vertebral level (*P* < .0001) was found for both volume (Figure 3A) and fat signal fraction (Figure 3B) measurements. The erector spinae was on average larger than the multifidus (125.8 vs 54.0 mL), and the level with the largest volume of muscle was found to be L3 (121.0 vs 28.9 mL − 115.0 mL). The volume of the multifidus was found to be significantly lower than the erector spinae from L1 to L4 (*P* < .0001), but significantly larger than the erector spinae at L5 and S1 (*P* < .0032). The overall fat signal fraction was found to be slightly higher in the erector spinae than the multifidus (0.228 vs 0.205), and the fat signal fraction increased from each level from L1 (0.188) to S1 (0.338). Multifidus fat signal fraction was found to be significantly higher than erector spinae at L1‐L3 (*P* < .0116), and significantly lower than erector spinae at L4‐S1 (*P* < .0001).

**Figure 4:**
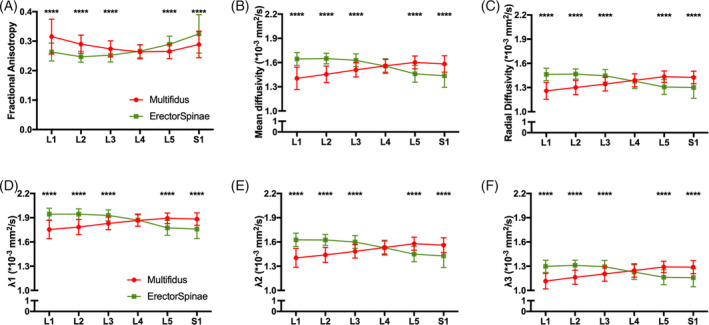
Diffusion tensor imaging measurements at each lumbar spine level (L1‐S1) for the multifidus (red) and erector spinae (green) muscles: A, Fractional anisotropy, B, mean diffusivity, C, radial diffusivity, D, *λ*1, E, *λ*2, and F, *λ*3. Asterisks indicate significant differences (*P < .05; **P < .01; ***P < .001; ****P < .0001)

For diffusion measurements, a significant difference between muscles (*P* < .0084) and a significant interaction effect between muscle and vertebral level was found for all variables (*P* < .0001; Figure 4). In addition, a significant effect of level was found for FA, MD, and *λ*_1_ (*P* < .0065), a trend was found for *λ*_2_ and *λ*_3_ (*P* = .0608, *P* = .0989), and a significant effect of level was not observed for RD (*P* = .1623). Overall, a less restricted diffusion profile was observed in the erector spinae compared to the multifidus, with the greatest overall diffusion found between the L2 and L4 levels. Interestingly, post hoc tests revealed significant differences of all diffusion measurements between the multifidus and erector spinae at all levels (*P* < .0001) except L4 (*P* > .2968). The magnitude of the difference between significant diffusion measurements ranged from 7% to 20%. Overall, diffusion was more restricted in the multifidus than the erector spinae above L4, and diffusion was more restricted in the erector spinae than the multifidus below L4.

Level dependent and total muscle volume, fat fraction, and restricted diffusion measurements for the erector spinae and multifidus are additionally reported in Table S1.

### 3.3 Qualitative assessment of 3D muscle size, fat fraction, and tractography

**Figure 5:**
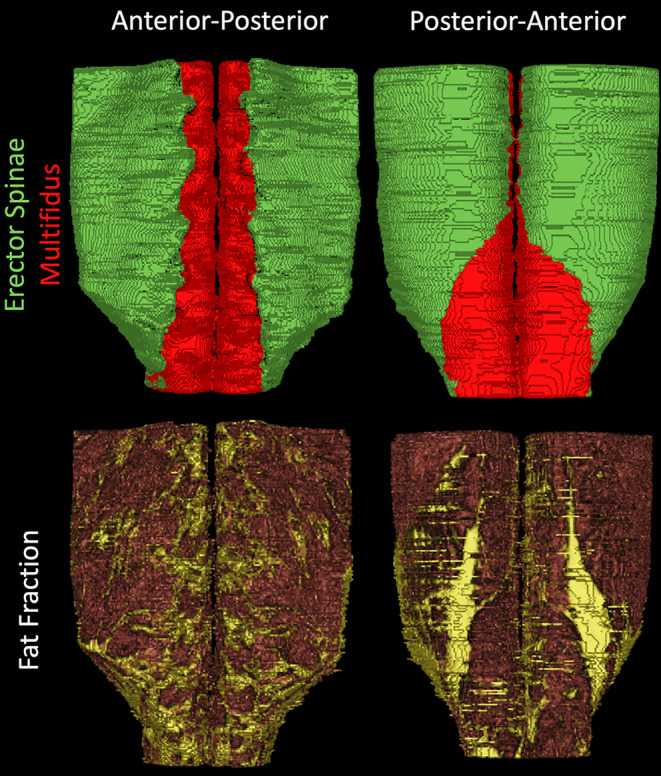
Three dimensional reconstructions of muscle volume (top) of the erector spinae (green) and multifidus (red) and fat signal fraction (bottom; muscle = red, fat = yellow) of the lumbar spine muscles from L1 to S1 in a representative subject

3D reconstructions of lumbar muscle size and quality visually depict the quantitative measures of size and fat fraction reported in Figure 3. The erector spinae inserts into the iliac crest, and the multifidus inserts into the vertebral lamina from the spinous and mammillary processes (Figure 5, Video S1). The top of the iliac crest is roughly at L4, and at or below this level, the erector spinae begins to lose volume. The multifidus muscle is smallest in the upper lumbar spine, and is largest below the iliac crest, at L4 and L5.

3D reconstructions of fatty infiltration within the lumbar muscles indicate increasing amounts of fat in the lower lumbar spine compared to the upper lumbar spine (Figure 5; Video S2). Additionally, in this highly active population, there is a lack of diffuse intramuscular fat, which is commonly observed in patients with pathology or low back pain^8^, ^9^, ^10^, ^33^, ^40^ (Video S3). Rather, the majority of fat in the muscles appears to be epimuscular; residing between epimysium and the fascial planes. In the anterior lumbar spine, there is a pronounced accumulation of fat in the medial multifidus muscle at every level, roughly where the paraspinal nerves innervate the lumbar musculature. Additionally, there is a distinct accumulation of epimuscular fat on the posterior aspect of the lumbar muscles, running from L3 to L5, which is not present in the superior lumbar spine, commonly referred to as a “fatty tent” in CSA studies of the lumbar spine.^33^, ^40^

**Figure 6:**
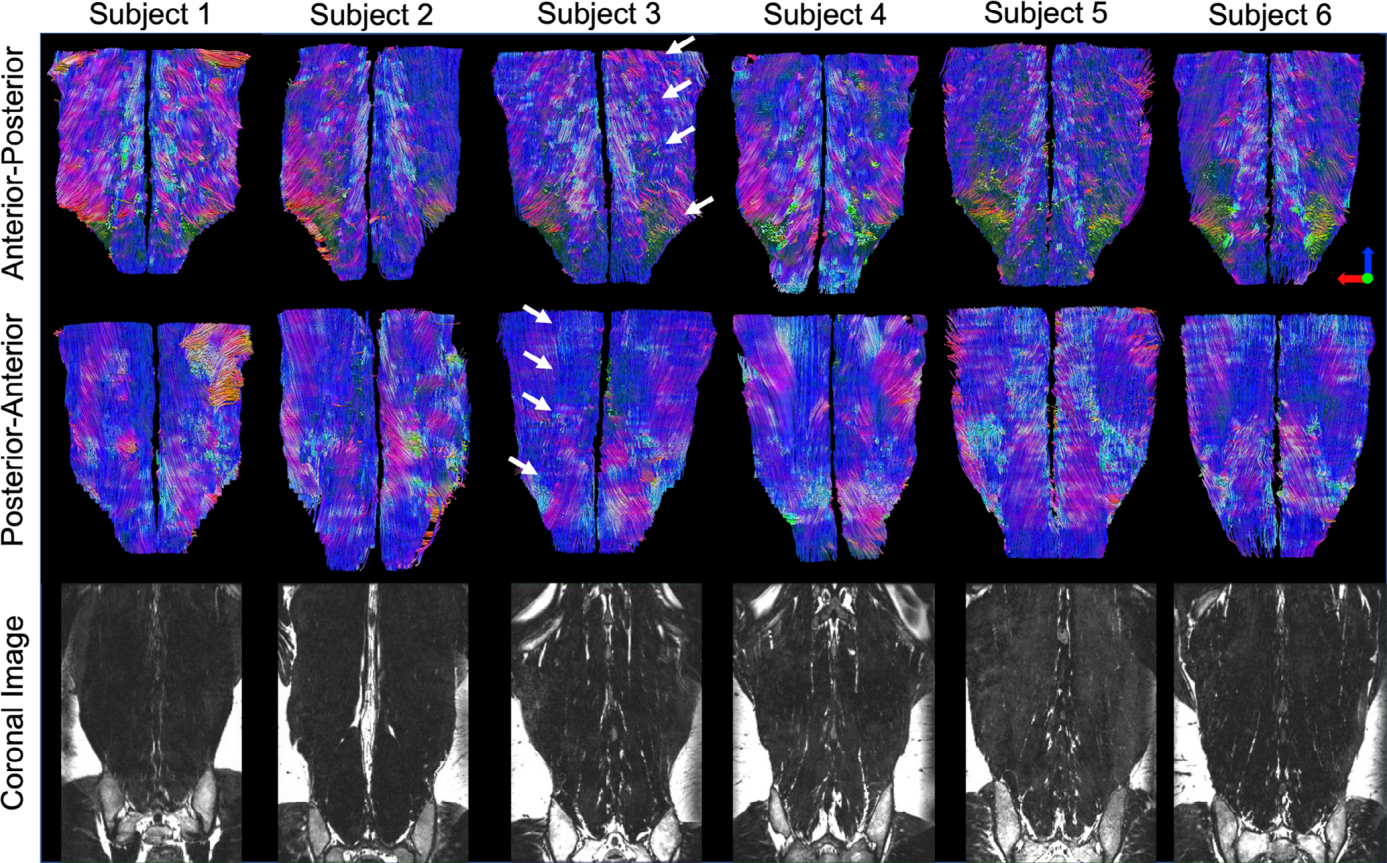
Representative tractography images of the lumbar spine from 6 Marines (Top, middle rows) with corresponding high resolution reformatted coronal anatomical MRIs (bottom row). Colors in the tractography scans indicate direction of the tracked fibers (blue—superior/inferior; red—medial/lateral; green anterior/posterior). These images demonstrate subject‐to‐subject variation in lumbar muscle size and fiber orientation. The border between the illiocostalis and longissimus muscles is highlighted by white arrows for one subject, which cannot be seen in the corresponding anatomical image

In order to demonstrate the repeatability of tractography, and its sensitivity to patient specific features of muscle architecture, sample tractography maps and their corresponding anatomical imaging is demonstrated from six subjects (Figure 6). Subject specific variations in muscle fiber orientation and gross muscle morphology are evident from 3D tractography models, that are supported by coronal reconstructions of high resolution anatomical imaging. Tractography models of the lumbar musculature demonstrate the pennation angle of the multifidus muscle, with fibers originating at the vertebral lamina and inserting into the spinous process, as compared to the erector spinae muscle, whose fibers originate at the iliac crest and are more oriented in an inferior‐superior direction. Additionally, due to their differing fiber orientations,^41^ the border between the longissimus and illiocostalis muscles of the lumbar spine is evident from tractography (Figure 6, arrows), which is traditionally difficult to identify with routine structural imaging. Finally, in the posterior‐anterior views of the lumbar muscle tractography, there are some fibers that appear to be out of the expected plane of muscle fiber direction, which likely represents muscle fibers inserting into the lumbosacral fascia.

## 4 DISCUSSION

In this study, we systematically measured lumbar muscle volume, fat signal fraction, and restricted diffusion in 42 active‐duty Marines. The erector spinae was larger than the multifidus above L5, had a lower fat signal fraction above L3, and had a less restricted diffusion profile than the multifidus above L4. However, this pattern was reversed in the lower lumbar spine, where the erector spinae was smaller than the multifidus, had a higher fat signal fraction, and demonstrated a more restricted diffusion profile.

A secondary goal of this study was to qualitatively investigate muscle composition in 3D. This allows for key properties of muscle to be visually identified, which is difficult to appreciate from quantitative measurements as they are averaged across the ROI and reduced to a single value. 3D reconstructions can be used to identify both epimuscular and intramuscular fat associated with muscle degeneration, which can be used to discern between muscle atrophy (associated with increased epimuscular fat), and fatty infiltration (associated with increased intramuscular fat). Additionally, 3D volumetric reconstruction of muscle quality can be used to investigate common features of fatty infiltration such as the epimuscular “fatty tent,” a deposit of fat which resides between the posterior aspect of the epimysium and the posterior fascial plane. From 3D reconstructions, it is evident that this “fatty tent” is mainly present in the lower lumbar spine, and dissipates in the upper levels. As the fatty tent is sometimes present in both highly active subjects and patients with pathology,^33^, ^40^ and the size of this tent varies within these populations, its effect on muscle function and lumbar muscle health is currently unknown and of current research interest.

Muscle tractography is a powerful post processing tool, which allows a user to approximate muscle fiber orientation in 3D. While tractography can be used to approximate fiber length, it is important to note that the size of a muscle fiber (~50 μm diameter) is several orders of magnitude smaller than the in plane resolution of a voxel (1.5 mm × 1.5 mm) and therefore does not actually measure true fiber size (or diameter). Previous studies have claimed that this technique can be used to measure physiologic cross sectional area (PCSA) of muscle.^20^, ^42^ PCSA is a measurement of muscle architecture, capable of predicting whole muscle force based on muscle size, pennation angle, and normalized fiber length.^43^ While muscle size and pennation angle can be obtained from DTI based tractography, normalized fiber length requires obtaining sarcomere length measurements, which to date can only be performed using invasive laser diffraction or polarized light microscopy techniques. Therefore, accurate PCSA measurements cannot be made using tractography.

One possible application of tractography is for muscle segmentation. Most muscles are manually segmented based on clearly defined fascial planes from routine or high‐definition anatomical imaging. However, for some muscles, such as the illiocostalis and longissimus muscles of the erector spinae, there is no clear fascial plane which can be used to discern the two. From tractography, it is evident that the fiber orientations between the illiocostalis and longissimus differ (Figure 6), and therefore may be useful for muscle segmentation. An additional application of tractography is the estimation of muscle fiber length in response to mechanical stimulation. A muscle fiber's force generating potential is related to its length.^44^ The ability to noninvasively assess muscle fiber lengthening (due to eccentric exercise^45^, ^46^) or shortening (due to chronic immobilization^47^, ^48^) provides valuable biomechanical information about a muscles potential force generating capacity, which may be of interest to both clinical and modeling communities.

The MRI protocol used in this study utilized a series of high resolution MRI scans to assess lumbar muscle characteristics of the entire volume of the lumbar spine in less than 1 hour. Additionally, this study detailed a series of standardized image analysis techniques based on easily identifiable features of muscle physiology in order to measure MRI based muscle size, fatty infiltration, and microstructure of the entire lumbar spine with minimal bias. Fascial planes were used as borders for ROI analysis, and the midpoint between adjacent vertebrae as the boundary for level based analysis. Previous studies utilizing DTI to measure lumbar spine microstructure have not used the entire muscle belly for analysis, but instead draw regions of interest in the center of the muscle on anatomical^26^, ^27^ or FA maps from the resulting diffusion data,^28^ which introduces bias into quantified diffusion measurements and does not measure diffusion data from the entire muscle. Finally, in order to remove susceptibility‐induced geometric distortions—common with DTI echo planar imaging sequences—the diffusion data was collected in both positive and negative phase encoding directions. This pair of images was used to estimate the susceptibility‐induced off‐resonance field,^36^ and then corrected and combined into a single image.^37^ While this doubled the scan time for the DTI acquisition, it ensured that the diffusion data could be accurately anatomically mapped.

The DTI sequence used in this study did not use fat suppression, which allowed adipose tissue within the muscle to contribute to the overall diffusion signal. Williams et al showed that a fat signal percentage of greater than 45% can significantly reduce overall diffusion in skeletal muscle.^49^ In this study, while there was no level with an average fat signal fraction greater than 45% for either muscle, ~13% of the individual voxels within the anatomic ROI did have fat signal fractions greater than 45%. A post hoc analysis was performed in order to determine if the differences in diffusion measurements between the erector spinae and multifidus muscles found in this study were driven by differences in fat signal fraction. A two‐way analysis of covariance (covariate: fat signal fraction) demonstrated the same significant main effects and post hoc comparisons as was tested using the two‐way repeated measures ANOVA analysis. Therefore, we concluded that it was unlikely that diffusion measurements were substantially affected by the relative low fat signal fraction measured in the lumbar musculature of these subjects.

The differences in lumbar muscle size and restricted diffusion in the upper and lower levels that were found may be reflective of the previously defined roles of these muscles to stabilize and provide motion of the spinal column. While the multifidus muscle has been identified as the primary stabilizer of the lumbar spine, the erector spinae also functions to stabilize the lumbar spine, as well as provide motion of the trunk. However, below L4, both the gross volume and restricted diffusion profile of the erector spinae suggests the multifidus is muscle larger at these levels. Therefore, the erector is likely providing little to no support below L4, making the multifidus the predominant muscle stabilizing the lower lumbar spine. Interestingly, the L4/L5 and L5/S1 motion segments are key structures of the spinal column, as well as the levels most implicated in failure^50^, ^51^ (ie, IVD degeneration, IVD herniation). A common radiological finding in patients with lower lumbar IVD injury is increased fatty infiltration of the multifidus muscle, which in turn reduces the functional contractile volume of the multifidus available to stabilize these lumbar levels. It is unclear if fatty infiltration is a precursor to instability/injury of the spinal column or whether it is a secondary consequence of injury. These findings supports current therapeutic strategies for patients with low back pain which target strengthening the multifidus, reducing fatty infiltration, and increasing muscle size in order to maintain stability of the lumbar spine.^22^, ^52^, ^53^, ^54^

There are a few limitations to this study. First, the Marines in this study are a highly‐active, male population, with no underlying pathology observable. Therefore these findings may only extend to a similar, highly‐active population. Second, the Marines in this study were not recruited based on history or presence of low back pain at the time of the study. Although this population is healthy and active, ~1/3 of the Marines included in the study reported having some history of low back pain. This is in agreement with a previous effort that evaluated combat injuries, where 33% of the respondents reported low back pain associated with their military occupation.^55^ However, this pain was not so severe that they were relieved of duty, or had seen a physician about the pain, and was most often attributed to training or combat demands.

## 5 CONCLUSION

In this study, level dependent differences in lumbar muscle size, fat signal fraction, and restricted diffusion were systematically measured in a group of active‐duty Marines. This is the first study to demonstrate that level dependent, MRI‐based microstructural differences exist between the erector spinae and multifidus muscles. Overall, the erector spinae was larger than the multifidus above L5, with lower fat signal fraction above L3, and a less restricted diffusion profile than the multifidus above L4. However, this pattern was reversed in the lower lumbar spine. Tractography of the lumbar musculature visually depicted patient specific variations in muscle architecture, as well as gross differences in the orientation of the multifidus and erector spinae muscles, which is often difficult to identify with routine structural imaging. The diffusion data from this study is representative of a highly active, healthy population. Future studies comparing these data to patients with low back may elucidate muscle microstructural changes associated with disease or pathology, which may affect the stability of the spine.

## CONFLICT OF INTEREST

The authors declare no competing conflict of interest.

## AUTHOR CONTRIBUTIONS

D.B.B. and A.E.R.S. contributed equally to this manuscript. Research design D.B.B., A.E.R.S., L.R.F., K.R.K., and S.R.W. Data acquisition A.E.R.S and D.B.B. Data analysis D.B.B., A.E.R.S., E.K.E., B.S., and C.P. Manuscript preparation D.B.B., A.E.R.S., E.K.E., and B.S. All authors have reviewed and approved the final submitted version of the manuscript.

### DISCLAIMER

I am a military service member or employee of the U.S. Government. This work was prepared as part of my official duties. Title 17, U.S.C. §105 provides that copyright protection under this title is not available for any work of the U.S. Government. Title 17, U.S.C. §101 defines a U.S. Government work as work prepared by a military service member or employee of the U.S. Government as part of that person's official duties.

Report No. 20‐06 was supported by U.S. Army Medical Research Acquisition Activity under work unit no. N1305. The views expressed in this article are those of the authors and do not necessarily reflect the official policy or position of the Department of the Navy, Department of Defense, nor the U.S. Government.

The study protocol was approved by the Naval Health Research Center Institutional Review Board in compliance with all applicable Federal regulations governing the protection of human subjects. Research data were derived from an approved Naval Health Research Center, Institutional Review Board protocol number NHRC.2013.0023 and University of California, San Diego protocol 110483.

## Supporting information


**Table S1** Volume, fat signal fraction, and restricted diffusion measurements for the erector spinae and multifidus muscles at each lumbar level

**Video S1**

**Video S2**

**Video S3**
