# Supplementary material for: Multiparametric MRI characterization of level dependent differences in lumbar muscle size, quality, and microstructure
Source: JOR Spine. 2020 Feb 3;3(2):e1079. doi: 10.1002/jsp2.1079 (PMC7323468; doi:10.1002/jsp2.1079)
Supplement: Supplementary file 1 — Table S1 Volume, fat signal fraction, and restricted diffusion measurements for the erector spinae and multifidus muscles at each lumbar level [file JSP2-3-e1079-s001.docx]

**Supplementary Table 1.** Volume, fat signal fraction, and restricted diffusion measurements for the erector spinae and multifidus muscles at each lumbar level

|  | Erector Spinae | | | | | | |
| --- | --- | --- | --- | --- | --- | --- | --- |
|  | L1 | L2 | L3 | L4 | L5 | S1 | total |
| **Volume (mL)** | 152.8±31.5 | 186.4±31.0 | 186.3±37.6 | 148.4±42.0 | 67.1±38.5 | 14.1±17.8 | 755.0±157.8 |
| **Fat Signal Fraction** | 0.153±0.032 | 0.164±0.044 | 0.199±0.056 | 0.244±0.067 | 0.317±0.078 | 0.410±0.093 | 0.201±0.047 |
| **FA** | 0.263±0.030 | 0.247±0.018 | 0.252±0.023 | 0.266±0.022 | 0.289±0.028 | 0.325±0.065 | 0.259±0.017 |
| **MD (*10^-3^ mm^2^/s)** | 1.645±0.080 | 1.650±0.066 | 1.628±0.079 | 1.556±0.091 | 1.460±0.104 | 1.436±0.143 | 1.610±0.058 |
| **RD (*10^-3^ mm^2^/s)** | 1.462±0.077 | 1.468±0.064 | 1.446±0.078 | 1.378±0.091 | 1.306±0.091 | 1.300±0.135 | 1.429±0.060 |
| **λ1 (*10^-3^ mm^2^/s)** | 1.945±0.074 | 1.944±0.066 | 1.928±0.069 | 1.871±0.076 | 1.775±0.091 | 1.759±0.118 | 1.912±0.056 |
| **λ2 (*10^-3^ mm^2^/s)** | 1.626±0.084 | 1.625±0.068 | 1.599±0.080 | 1.529±0.093 | 1.450±0.094 | 1.428±0.144 | 1.584±0.063 |
| **λ3 (*10^-3^ mm^2^/s)** | 1.298±0.075 | 1.311±0.063 | 1.293±0.078 | 1.228±0.090 | 1.163±0.093 | 1.158±0.112 | 1.274±0.059 |
|  | **Multifidus** | | | | | | |
|  | **L1** | **L2** | **L3** | **L4** | **L5** | **S1** | **total** |
| **Volume (mL)** | 25.4±4.3 | 36.8±8.4 | 55.7±10.4 | 81.6±13.6 | 80.5±15.7 | 43.8±15.0 | 323.9±48.4 |
| **Fat Fraction** | 0.224±0.047 | 0.215±0.053 | 0.209±0.052 | 0.215±0.056 | 0.243±0.062 | 0.265±0.058 | 0.228±0.052 |
| **FA** | 0.315±0.059 | 0.290±0.031 | 0.274±0.027 | 0.264±0.024 | 0.265±0.025 | 0.289±0.045 | 0.278±0.023 |
| **MD (*10^-3^ mm^2^/s)** | 1.404±0.139 | 1.455±0.103 | 1.509±0.088 | 1.559±0.080 | 1.601±0.079 | 1.582±0.101 | 1.539±0.060 |
| **RD (*10^-3^ mm^2^/s)** | 1.259±0.105 | 1.301±0.088 | 1.344±0.086 | 1.389±0.081 | 1.435±0.071 | 1.424±0.078 | 1.376±0.057 |
| **λ1 (*10^-3^ mm^2^/s)** | 1.754±0.114 | 1.785±0.095 | 1.830±0.079 | 1.867±0.071 | 1.894±0.067 | 1.883±0.078 | 1.851±0.054 |
| **λ2 (*10^-3^ mm^2^/s)** | 1.403±0.117 | 1.439±0.093 | 1.483±0.083 | 1.532±0.081 | 1.579±0.081 | 1.560±0.091 | 1.515±0.058 |
| **λ3 (*10^-3^ mm^2^/s)** | 1.115±0.096 | 1.163±0.086 | 1.204±0.090 | 1.247±0.084 | 1.291±0.071 | 1.288±0.080 | 1.238±0.061 |

*Abbreviations: FA – Fractional Anisotropy; MD - Mean Diffusivity; RD – Radial Diffusivity*
